# Design and validation of fiber optic localized surface plasmon resonance sensor for thyroglobulin immunoassay with high sensitivity and rapid detection

**DOI:** 10.1038/s41598-021-95375-y

**Published:** 2021-08-06

**Authors:** Hyeong-Min Kim, Dae Hong Jeong, Ho-Young Lee, Jae-Hyoung Park, Seung-Ki Lee

**Affiliations:** 1 Department of Electronics and Electrical Engineering, Dankook University, Yongin, 16890 South Korea; 2 Department of Chemistry Education, Seoul National University, Seoul, 08826 South Korea; 3 Department of Nuclear Medicine, Seoul National University Bundang Hospital, Seongnam, 13620 South Korea

**Keywords:** Biosensors, Nanophotonics and plasmonics, Nanotechnology in cancer, Microfluidics, Optical spectroscopy

## Abstract

A simple optical fiber sensor based on localized surface plasmon resonance was constructed for direct and rapid measurement of thyroglobulin (Tg). Specific tests for Tg in patients that have undergone thyroidectomy are limited because of insufficient sensitivity, complicated procedures, and in some cases, a long time to yield a result. A sensitive, fast, and simple method is necessary to relieve the psychological and physical burden of the patient. Various concentrations of Tg were measured in a microfluidic channel using an optical fiber sensor with gold nanoparticles. The sensor chip has a detection limit of 93.11 fg/mL with no specificity for other antigens. The potential applicability of the Tg sensing system was evaluated using arbitrary samples containing specific concentrations of Tg. Finally, the sensor can be employed to detect Tg in the patient’s serum, with a good correlation when compared with the commercial kit.

## Introduction

Differentiated thyroid cancer (DTC) is the most common endocrine cancer, with an increasing incidence worldwide^1^. Although DTC progresses slowly and has low mortality, the incidence of recurrence or residual cancer after initial treatment, usually with total thyroidectomy, is high^2^, which is associated with increased long-term morbidity^3^. Thus, life-long follow-up is recommended for early determination of recurrence or residual cancer to prevent cancer progression and death as well as the unnecessary examination of patients with a low risk of recurrence^4^. Thyroglobulin (Tg) is a 660 kDa dimeric glycoprotein produced by the thyroid follicular cells. It is a precursor protein, essential for thyroid hormone synthesis and is also measured in the serum of healthy individuals. The serum concentration represents the amount of existing differentiated thyroid tissue, damage to thyroid tissue, and the amount of thyroid globulin excretion because of inflammation, thyrotropin (TSH), human choriogonadotropin, and TSH receptor stimulation such as TSH receptor antibodies. Tg has high specificity as a DTC biomarker^5^. Because Tg is secreted only from normal thyroid tissue and thyroid cancer tissue, its measurement helps in the early detection of recurrent or residual thyroid cancer in patients that undergo total thyroidectomy^6^.

Tg detection has been conventionally performed using techniques that fix primary antibodies to solid matters such as test tubes or beads, and then introduce additional secondary antibodies to enhance sensitivity; this requires highly skilled personnel and is complex and time-consuming^7^. When Tg measurement was performed using an immunoradiometric assay (IRMA), immunomatric enzyme assay (IeMA), or chemiluminescence assay (CLIA), the limit of detection (LOD) of IRMA, IeMA, and CLIA was 0.01 ng/mL. To overcome the limited sensitivity, the patient should suspend thyroxine for 2–4 weeks before Tg measurement^8^. Furthermore, the consumption time was 1–24 h, which could be increased in relation to the number of measurement samples. The measurement of serum Tg levels is crucial for DTC management. Therefore, it is necessary to develop biosensors capable of evaluating Tg in a rapid, sensitive, and preferably label-free manner^9^.

Localized surface plasmon resonance (LSPR) is a phenomenon in which light resonates with the collective oscillation of free electrons on the surface of nanoparticles when they enter the noble metal nanoparticles smaller than the wavelength of the incident light^10^. LSPR biosensors that depend on the position or intensity of the resonance according to the refractive index around the nanoparticles have been widely studied with advantages such as a fairly simple method, high sensitivity, and real-time detection^11,12^. Generally, the biomolecules contained in the buffer solution have a larger refractive index than the buffer solution, and their adsorption or dissociation onto the noble metal nanoparticles causes a refractive index change around the nanoparticles^13^. Recently, fiber optic LSPR (FO LSPR) sensors have been developed by immobilizing nanoparticles on optical fiber, based on a simple optical set-up^14,15^; this has the potential of rapid detection, low-cost, portability, and simplicity when applied to Tg sensing.

In this study, an FO LSPR sensor chip is proposed by immobilizing gold nanoparticles (AuNPs) on an optical fiber and combining it with a microfluidic channel (MFC) that provides a continuous fluid without exposing it to the external environment. MFC improves reproducibility in measurement by preventing the evaporation of biomolecule solutions and the loss of AuNPs on the optical fiber surface, which can be caused by exposure of the sensor tip to air^16^. The fabricated sensors were applied to detect various concentrations of Tg, which is a useful biomarker for monitoring the recurrence of thyroid cancer. The detection time per sample was 10 min. The FO LSPR sensor chips exhibit a good correlation between the changes in LSPR intensity and the concentrations of Tg, as well as negligible intensity shifts for another antigen. Additionally, when the arbitrary concentrations of unknown levels to the tester were measured and the values of Tg were estimated based on the standard curve, the proposed sensor exhibited a low error rate compared with the actual concentrations. Finally, Tg in the serum of patients was quantified, and the results were compared with those of commercial IRMA kits. The developed sensors exhibited a high correlation with the devices used in the clinical field. These results show that our sensor can be a helpful tool for the early evaluation of the recurrence of thyroid cancer through sensitive and quick sensing of Tg in patients that undergo total thyroidectomy.

## Materials and methods

### Materials

All research was performed in accordance with the principles of the Helsinki Declaration as revised in 2013. Multimode optical fiber (105 μm core, FG105LCA) was purchased from Thorlabs, USA. Slide glass (size: 7.6 × 2.6 × 0.1 cm) was supplied by Duran, Germany. Tygon tubes (internal diameter, 0.51 mm; external diameter, 1.52 mm; S54-HL) were acquired from Hanmi Rubber and Plastics, South Korea. A polydimethylsiloxane (PDMS) base and curing agent were provided by Dow Corning, USA. Sulfuric acid (95%) and isopropanol (99.5%) were purchased from Daejung Chemicals, South Korea. Hydrogen peroxide (34.5%) was obtained from Samchun Chemical, South Korea. 3-(Ethoxydimethylsilyl)propylamine (APDMES, 97%) and bovine serum albumin (BSA, ≥ 98%) were gained from Sigma Aldrich, USA. Borate buffer (0.05 M, pH 8.5) was purchased from Bioworld, USA. Anti-Tg antibody, Tg, and Alpha-fetoprotein (AFP) were provided by Shin Jin Medics, South Korea. Ferritin (from human spleen, P1450) was obtained in BioVision Inc., USA. The institutional review boards of Seoul National University Bundang Hospital approved this study and exempted the need for individual informed consent (IRB no. B-1711/432-302). Ten patients’ sera with different level of thyroglobulin were used for the study.

### Preparation of the sensing element

The fabrication steps of the FO LSPR sensor chips consist of three stages: (1) preparation of FO LSPR sensors, (2) production of MFCs, and (3) assembly of FO LSPR sensors and MFCs. In the first step involved in the manufacture of the FO LSPR sensors, the optical fibers were cut flat for stable transmission and collection of light using a fiber cleaver (S325A, Fitel, USA). The optical fibers which are cut by the cleaver was used without further polishing process^17^. Next, the cross-section of the fibers was treated in a Piranha solution (a mixture of 3/1 (v/v) sulfuric acid and hydrogen peroxide) for 20 min to remove the organic contaminants and activate the hydroxyl group for fixing the self-assembled monolayer (SAM). To form SAMs containing amino groups for immobilizing negatively charged AuNPs^18^, the fibers were reacted with 5% APDMES in isopropanol for 90 min. The fabrication of the FO LSPR sensors was completed by incubating the tips in a gold colloidal solution for 60 min. The gold colloid was prepared by the Turkevich method using deionized (DI) water, gold chloride, and sodium citrate^19^. The 0.01% gold in aqueous chloride and 1% sodium citrate aqueous solution were mixed with a volume ratio of 100:1. This condition guarantees a nanoparticle size of around 55 nm. It has been confirmed in a previous study that the FO LSPR sensor which is fabricated with nanoparticles around 55 nm and immobilization time of 60 min in colloid ensured good reproducibility between sensors in our optical set-up^20^. We measured the UV–Vis Spectrum (UV1800, Shimadzu, Japan) of the synthesized colloidal solution of AuNPs (Fig. S1).

In the second stage, MFCs are prepared to avoid the evaporation of biomolecules on the optical fiber surface, to avert degradation of sensor performance owing to the loss of AuNPs caused by frequent in-outs into the solutions, and to provide a continuous measurement environment^21^. The MFC has four inlets supplying different reagents, such as DI water, antibodies, blocking materials, antigens, and one outlet, which serves as a reaction chamber. The height and width of the channel were designed as 250 µm considering the diameter of the optical fiber containing the jacket, and the distance from the center of the inlet to the center of the outlet was approximately 12 mm. The silicon mold used to print the MFCs based on PDMS is simply fabricated by photolithography and deep reactive ion etching (DRIE)^22^. The polymer was deposited on a silicon mold using C_4_F_8_ gas in a DRIE machine (SLR-770-10R-B, Plasma Therm, USA) for weak adhesion with PDMS^23^. A mold based on a silicon wafer has little impact on the sensor manufacturing costs because it can be used semi-permanently. MFCs were formed by pouring the PDMS solution (a 10/1 (w/w) ratio of PDMS base to curing agent) into the mold, curing for 60 min in an 80 °C oven, and detaching the hardened PDMS from the mold. This series of processes is well-known as soft lithography^24^. Subsequently, the inlets and outlet were formed using a 1.5 mm diameter punch (Ted Pella, USA). A detail on the preparation process of MFC is provided in Fig. S2 of Supplementary Information.

In the third step, the PDMS channel was cut to 13 × 18 mm and then treated with oxygen plasma (CUTE, Femto Science, South Korea) for 50 s at 60 W, 50 kHz, and 0.1 Torr for bonding to the substrate^25^. Commercial slide glass was used as the substrate and it was attached to the MFC after the end face of the FO LSPR sensor was loaded into the reaction chamber of the MFC. The sensor chips were stored for 60 min at 80 °C in an oven to promote adhesion. The fabrication of the FO LSPR sensor chip was completed by connecting the tubes to the inlets and outlet for the supply and collection of fluids.

## Experimental set-up

**Figure 1:**
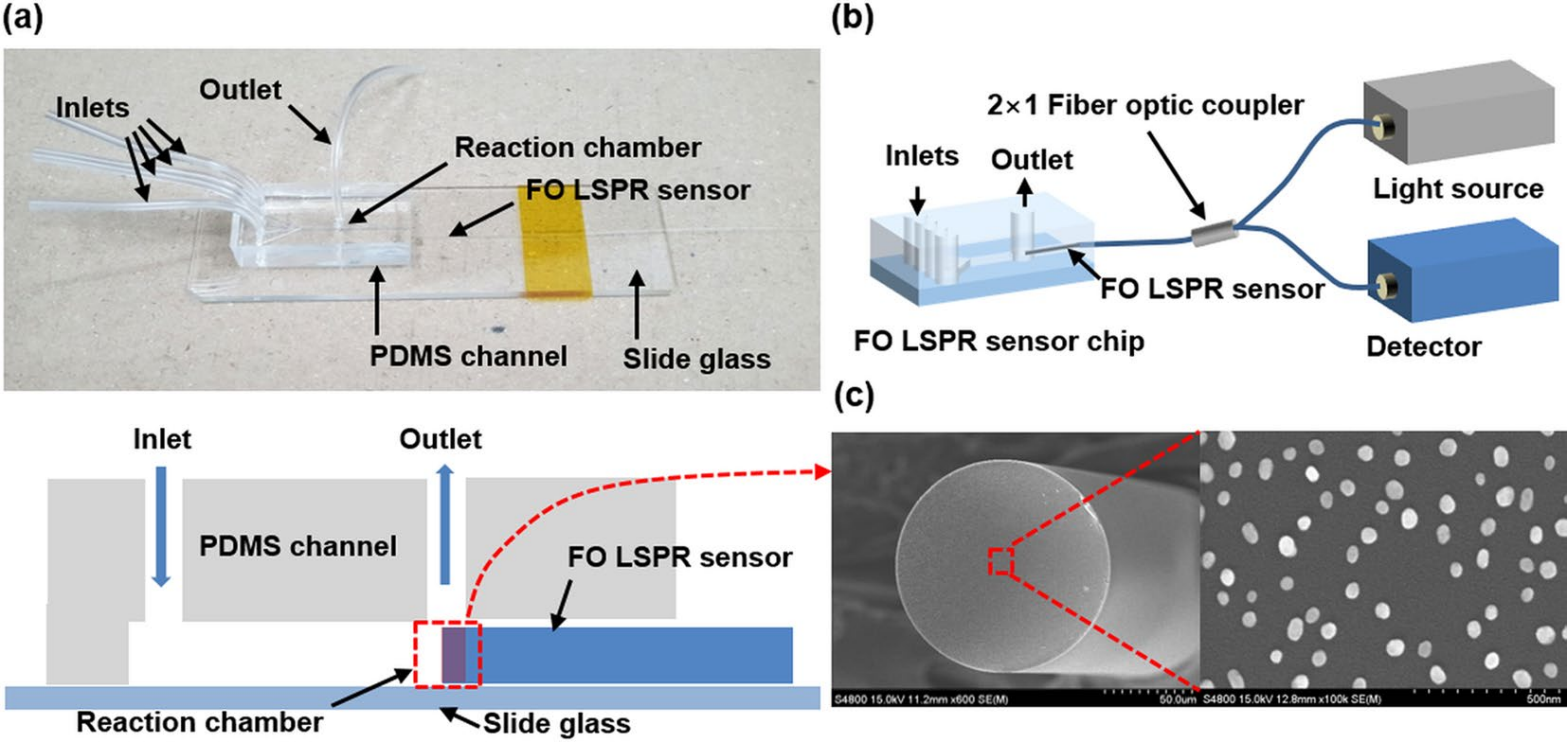
Fabricated FO LSPR sensor chip, optical set-up, and photograph of the sensor surface: (**a**) real picture of the sensor chip and side view, (**b**) simple optical measurement set-up, and (**c**) FE-SEM images of optical fiber end-facet with AuNPs.

For LSPR measurements, the sensor chip (Fig. 1a) was spliced into the optical set-up shown in Fig. 1b. The upper part of Fig. 1a is a photograph of the FO LSPR sensor chip, and the lower image is a sectional view of the sensor chip. In Fig. 1b, the simple optical system consists of a light source, a detector, and a 2 × 1 fiber coupler (Thorlabs). The one-port end of the coupler was connected to the sensor chip. The two-port ends were joined to a light source and detector, respectively. Light from the source propagates through the coupler to the surface of the optical fiber, and the resonance signal gained from the sensor is collected by the detector. White light (LS-1, Ocean Optics, USA) and a spectrometer (SM200, Spectral Products, USA) were used as the light source and detector for observing the LSPR spectra derived from the fabricated sensor, respectively; a laser (iFLEX-2000, Qioptiq, UK) and photodetector (PDA36A-EC, Thorlabs) were used for real-time monitoring of the sensorgram during the antibody-antigen reaction.

The tubes that are inserted into the sensor chip are linked to shut-off valves (P732, Revodix, South Korea) to prevent the backflow of reagents; the chemicals were sequentially supplied to the chip by syringe pumps (NE-300, New Era Pump Systems, USA). Each solution was supplied at a flow rate of 10 µL/min to improve biomolecule binding by reducing the bubbles generated in the channel, and to guarantee the stability of the AuNPs on the fiber surface with a small pushing force^26^. The diameter of the one milliliter syringe (Norm-Ject, Henke Sass Wolf, Germany) was 4.699 mm.

### Sensor testing

**Figure 2:**
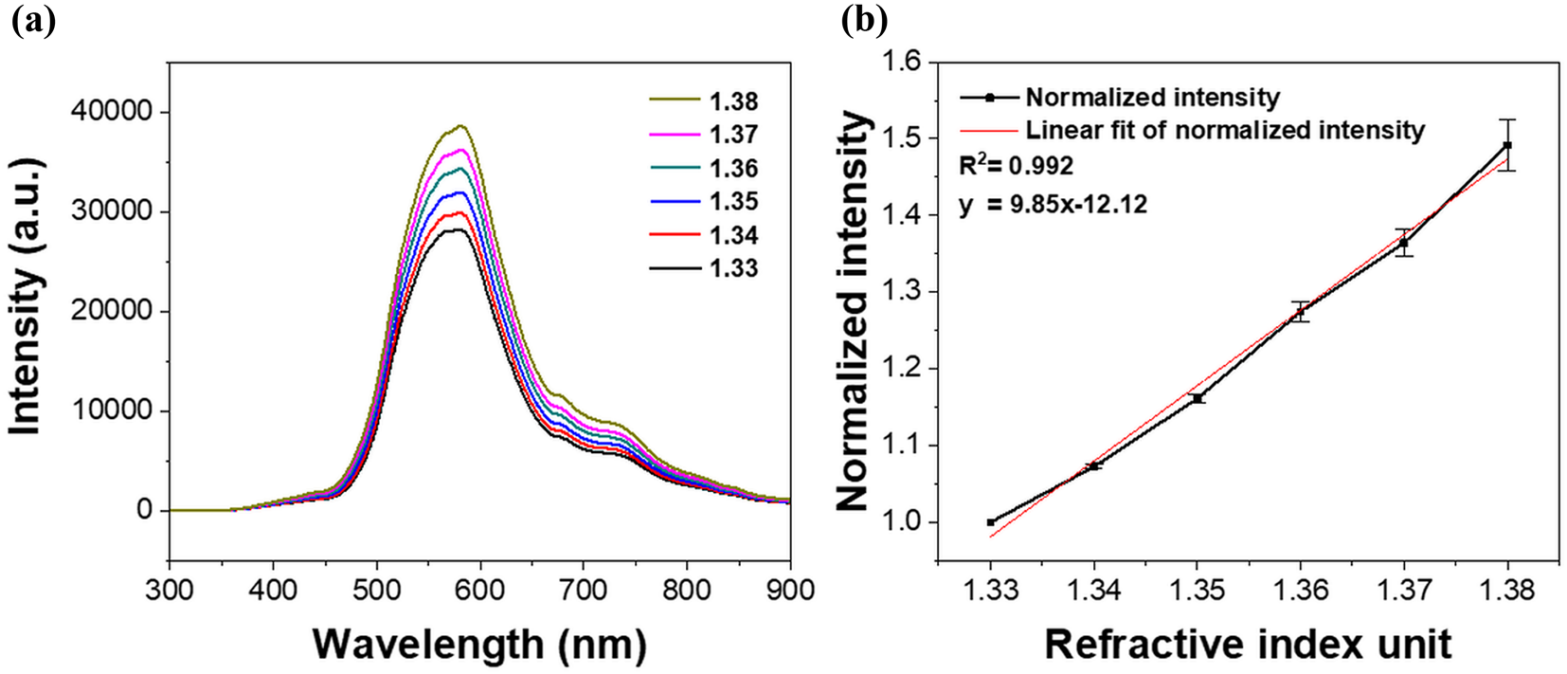
Refractive index characteristics of proposed sensor chip: (**a**) response of FO LSPR sensor for various refractive indices and (**b**) changes of LSPR intensity at 640 nm; all points are normalized by 1.33.

In Fig. 1c, the diameter and distribution of the AuNPs, which were immobilized on the cross-section of the optical fiber, were observed by field emission scanning electron microscopy (FE-SEM, S4800, Hitachi, Japan). The images confirmed the formation of monodispersed nanoparticles with an average size of 51.79 ± 5.68 nm. The sensor outputs were recorded in a series of solutions with various refractive indices to test the refractive index sensitivity of the proposed FO LSPR sensor chip. Figure 2a shows the measured spectra in the sensor chip when solutions with different refractive indices (1.33 to 1.38) are injected into the sensor tip. As the refractive index around the AuNPs increased, the LSPR intensity gradually increased. The LSPR spectra was recorded with the integration time of 11 ms. The position of the peak is slightly different between the FO LSPR sensors which are fabricated in one batch because nanoparticles are randomly adsorbed depending on the optical fiber. If the experiments are performed based on a different wavelength position each time, the reproducibility of the measurements may be decreased. Therefore, it can be advantageous to use other wavelengths than the peak wavelength^27^. The immunoassay was carried out at the same wavelength each time and the laser had a wavelength of 640 nm. 640 nm is a relatively long wavelength compared to the peak wavelength of 580 nm in the 1.33 refractive index. We respectively confirmed the responses to the refractive index change at relatively long wavelength (640 nm) and short wavelength (520 nm) based on the peak wavelength (Fig. S3). At this time, the outputs were normalized for comparison of response rates. Since a comparatively high response rate was recorded at a relatively long wavelength of 640 nm, we measured the antibody-antigen reaction using a laser having a longer wavelength than the resonance peak. Figure 2b shows a graph of the change in the output intensities at the 640 nm, which was fitted with a linear equation to study the slope and coefficient of determination (R^2^). In the y-axis, the intensities were normalized to perceive relative changes by dividing all the values using the output obtained at 1.33^28^. The slope and R^2^ indicate a strong correlation between the sensor outputs and variations in the refractive index. R^2^ is a statistical measure of the closeness of the data to the fitted regression line^29^. The experiment was repeated three times and the average of the coefficient of variations (CVs) was 1.03% at the five refractive indices except for the 1.33 refractive index. These results indicate that the fabricated sensor chip can be utilized as a linear refractive index sensor.

### Production of nanoparticle-antibody conjugates

**Figure 3:**
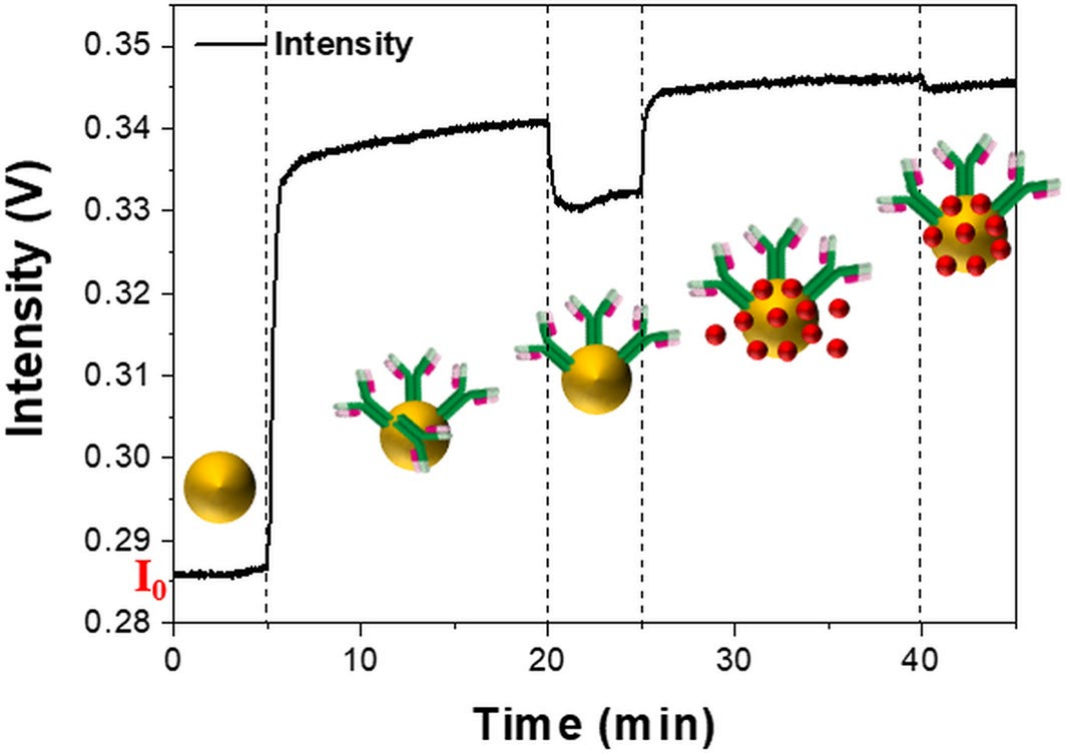
Sensorgram measured in real-time during attachment of antibodies to AuNPs.

The protocol for generating nanoparticle-antibody conjugates for Tg detection was performed in five steps, as shown in Fig. 3, and the entire process was performed within the channel. First, the resonance intensity, which was used as the reference signal (I_0_), was measured in DI water. I_0_ was recorded for at least 5 min after the stability of the output fluctuation. A high flow rate of 100 µL/min was used at the start to quickly fill up the fluidic channel; however, the reactions with reagents were carried out at a flow rate of 10 µL/min. In the second stage, 20 µg/mL Tg antibodies in the borate buffer were pumped for 15 min to immobilize the antibodies on the nanoparticles through an electrostatic bond between the negative charges on the surface of the AuNPs and the amino groups of the antibodies^30^. At this time, an increase in the LSPR intensity was observed, which was estimated due to the introduction of antibodies on the AuNPs. The residual antibodies were then washed with DI water for 5 min. Subsequently, a slight decrease in the sensor output was observed. In the fourth step, a 1% aqueous solution of BSA, well known as a blocking agent, was fed for 15 min to prevent nonspecific binding, except for the antibody-antigen reaction^31^. Finally, the sensor surface was rinsed for 5 min to eliminate excess BSAs. A total of 45 min was required to make the nanoparticle-antibody conjugates. To confirm the attachment process of the antibodies and BSAs, changes in the LSPR signals during different reaction stages were observed in real-time, as shown in Fig. 3. Each reaction procedure with antibodies and BSAs gradually increased the resonance intensity. By checking the net changes in the LSPR responses in DI water before and after the reaction steps, it was deduced that the antibodies and BSAs were well adsorbed on the surface of the AuNPs.

## Results and discussion

### Tg detection

**Figure 4:**
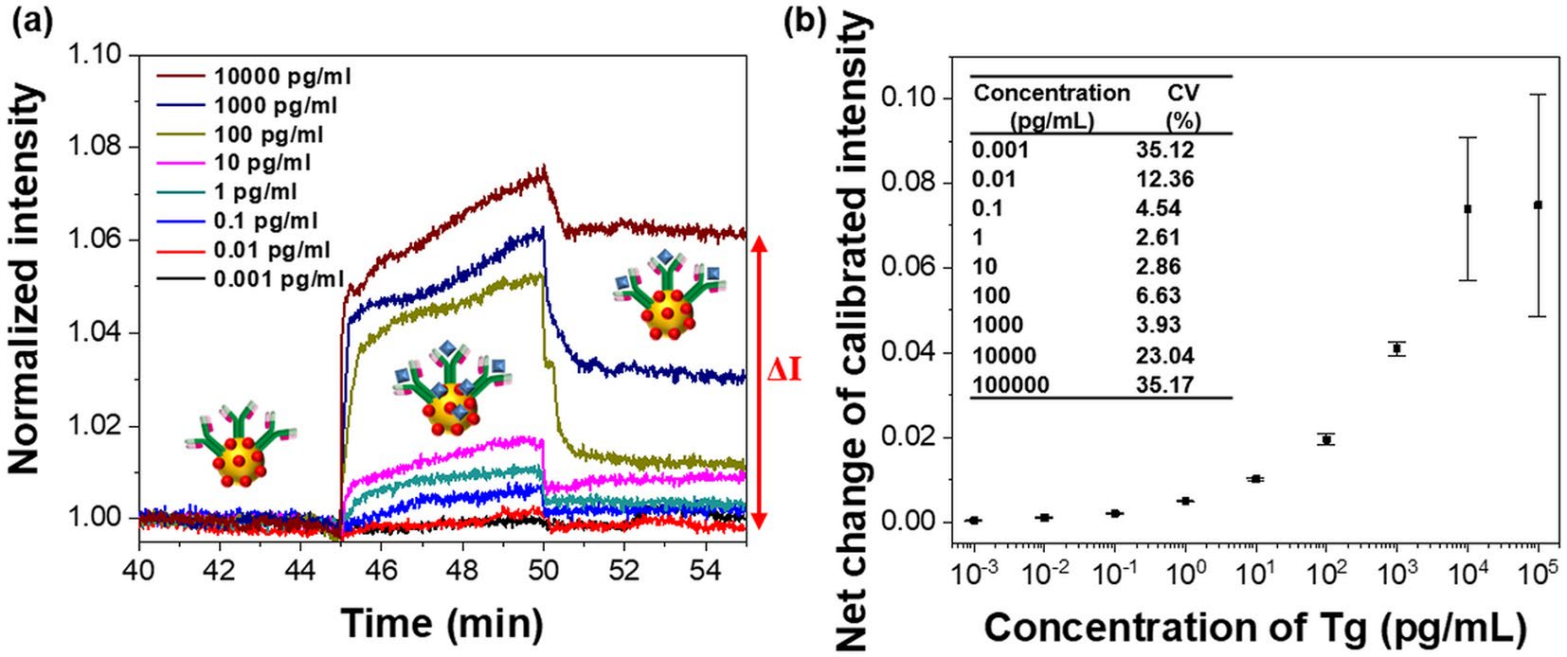
Results of Tg detection in various levels: (**a**) sensorgram recorded in real-time and (**b**) ΔI measured three times at each concentration and CVs (CV = (standard deviation/mean) × 100%).

Different Tg concentrations were measured to determine whether the sensor could be used for Tg detection. After the five steps in “Materials and methods” section, antibody-antigen interactions were induced for 5 min. Tg detection was then completed in approximately 10 min by supplying the water for 5 min to wash the unbound antigens, as shown in Fig. 4a. In the Tg range of 0.001–100,000 pg/mL, the differences in LSPR intensities before and after the antibody-antigen reactions obtained by measuring each concentration three times with different sensors are shown in Fig. 4b. In this graph, the net changes in intensity before and after the antibody-antigen interactions were divided by the reference signals (I_0_) acquired in each sensor, which were labeled as the calibrated intensity on the y-axis. We took a cross-sectional photograph of the optical fiber before measurement and obtained a picture of the fiber optic surface again after antibody-antigen reaction (Fig. S4). Also, the distribution of the nanoparticles before and after the experiment was shown as the histogram. As a result, it was confirmed that the particles were evenly distributed on the surface of the optical fiber regardless of the use. This is because the stability of nanoparticles on the optical fiber was secured in an MFC with separation for the external environment.

The I_0_, which was first recorded with only AuNPs in the DI water, was slightly different for each sensor. The magnitude of I_0_ tended to affect the differences between the signal intensities before and after the antibody-antigen reaction. Usually, when I_0_ is large, the signal changes according to the Tg binding are also measured with large values. Although the FO LSPR sensors were fabricated in one batch, the size, density, and position of the AuNPs immobilized on each optical fiber surface cannot be identical, which is a significant factor that affects the magnitude of I_0_. To uniformly control the characteristics of the FO LSPR sensors, the I_0_, which quantitatively shows the state of the nanoparticles on the optical fiber-based sensor surface, was measured at each sensor after sensor fabrication and used as a calibration signal for the results of the antibody-antigen interactions. There may be other ways to compensate for ΔI; however, we deduce that the calibrated results divided by I_0_ are sufficient with a simple method.

In Fig. 4b, a clear sigmoidal dose–response curve was observed, and the average of the CVs at all data points except the highest and lowest concentrations was 8.00%, which indicated high reproducibility of the results measured by the proposed sensor. A method that determines the LOD by converting the intensity value of the y-axis to the concentration of the x-axis using a regression line is proposed because the intensity levels are commonly obtained instead of the concentration in the analytical devices^32^. When the LOD was calculated by substituting three times the standard deviations into a linear equation-based regression line in the straight part of the graph, the LOD was 93.11 fg/mL. As biochemicals are introduced into the sensor chip, the response is initiated at the minimal effective dose and increases in intensity as the dose is increased. The dose ultimately reaches the point where no further increase in response is observed, which is referred to as the maximal dose^33^. The proposed sensor was considered to have a maximal dose of 10,000 pg/mL, thus, its dynamic range was 0.1–10,000 pg/mL. These results suggest that the FO LSPR sensor chip can be used as a highly sensitive instrument for determining DTC recurrence. The standard range of Tg in human serum is 0–60,000 pg/mL, which is because Tg is also produced in normal thyroid tissue. However, it is important to monitor Tg concentrations of the range below 10 ng/mL in patients who have undergone thyroidectomy or isotope therapy with thyroid cancer^34^. Long-term monitoring and follow-up of disease are essential to reduce patient mortality. The Tg level of less than 1 ng/mL which is measured between 6 months and 1 year after treatment can indicate a lower risk of recurrence later than 5 years at that time. At the time, simple cares such as taking medication and annual neck ultrasonography are fulfilled. At a Tg above 1 ng/mL, the patients stop thyroxine (T4) therapy and repeat Tg testing until the value drops below 1 ng/mL. If the Tg is more than 10 ng/mL, chest computed tomography or positron emission tomography is performed and patients with relapse suffer reoperation or radioactive iodine therapy. Therefore, the detection of Tg in the follow-up of patients with DTC is significant in the level below 10 ng/mL in particular.

To evaluate the specification of the proposed FO LSPR biosensor, sensing parameters such as LOD, detection range, and reaction time with antigen were compared with other Tg detection methods. Like listed in Table 1, the proposed FO LSPR system combined with MFC demonstrated lower LOD, wide detection range, and rapid detection time which were comparable or better than other methods.

**Table 1:** A comparison of sensing parameters between the Tg detection methods.

Method	LOD	Detection range	Reaction time with antigen (min)	References
Capillary electrophoresis	7.6 pg/mL	10 pg/mL–9.70 ng/mL	20	^35^
Long period grating	80 pg/mL	0.1 ng/mL–20 ng/mL	60	^36^
Fluorescence	4.2 pg/mL	10 pg/mL–1 ng/mL	60	^37^
Luminescence	18 fg/mL	0.1 pg/mL–15 pg/mL	60	^38^
LSPR	93 fg/mL	0.1 pg/mL–10 ng/mL	5	This work

**Figure 5:**
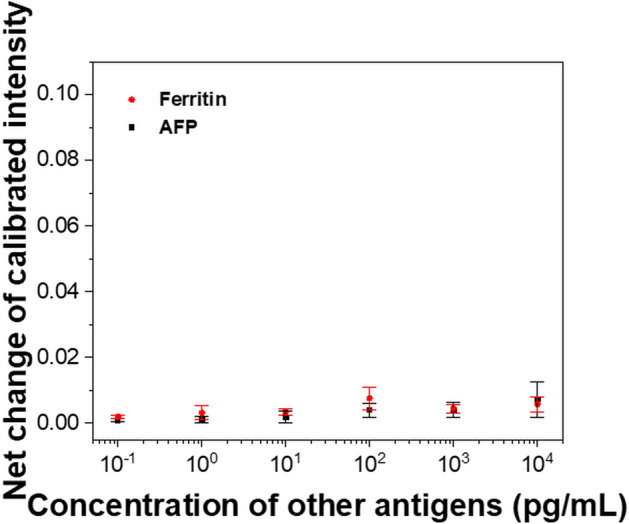
Selectivity of the FO LSPR sensor chip. Various levels of other antigens were measured by sensors fixed with Tg antibodies.

Figure 5 shows the selectivity of the proposed sensor to which the Tg antibodies were immobilized. AFP with the same concentrations as the dynamic range was reacted with the sensor on which the Tg antibodies were conjugated. AFP is used as a biomarker to diagnose and detect liver cancer, and its levels are also affected by thyroid hormones and hypothyroidism^39^. When various values of AFP were injected into the sensor chip, the changes in the LSPR intensities exhibited by the proposed sensor were minimal at all levels. This shows that the FO LSPR sensor chip can be configured to selectively detect Tg. AFP with a relatively small molecular weight of 70 kDa can output a lower difference of LSPR intensity when inducing nonspecific adsorption at the same concentration compared to other antigens with similar mass to Tg of 660 kDa. Therefore, we added nonspecific adsorption by ferritin with a molecular weight of 450 kDa. Ferritin is a universal intracellular protein responsible for storing iron in the body and its concentration is affected by the thyroid status. In hypothyroidism, ferritin level is low. On the other hand, ferritin concentration is elevated in hyperthyroidism. As a result, the proposed FO LSPR biosensor for Tg detection did not also output a specific trend depending on the antigen concentration even when the molecular weight of the antigen was relatively large.

### Blind test

**Figure 6:**
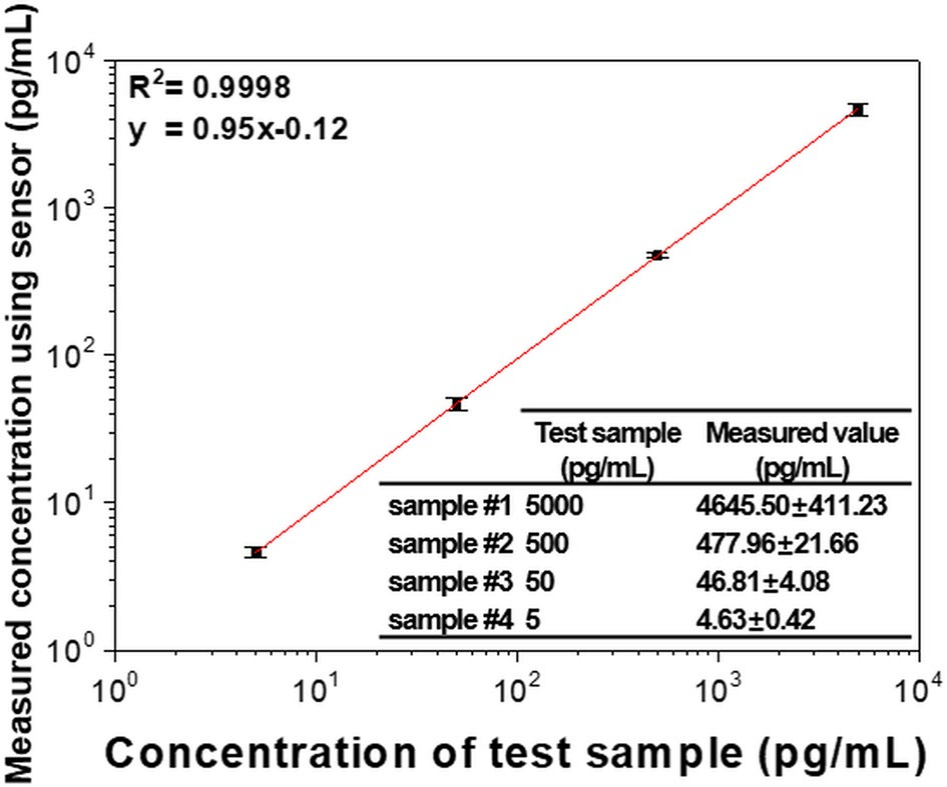
Estimation of Tg in the test samples with concentrations unknown to the tester.

We estimated arbitrary Tg levels using a standard curve based on Fig. 4b. Four test samples with unknown concentrations were prepared and measured three times at each point. The measured values were converted into concentrations using a standard curve based on a linear equation, as shown in Fig. 6. The x-axis of the graph represents the actual Tg concentration of the randomly prepared test samples, and the y-axis is the value measured by the proposed sensor. When the data of the graph were fitted by a linear equation, the slope was close to 1, and the R^2^ value indicating the correlation between the values of the x- and y-axis was 0.9998. The error between the measured values and the actual concentrations of the test samples was an average of 6.33%. The exact values of the actual and measured levels are shown in the inset. These results confirm that the proposed sensor can evaluate the actual Tg concentrations with high accuracy and has the potential for application as a diagnostic device.

### Serum sample testing

**Figure 7:**
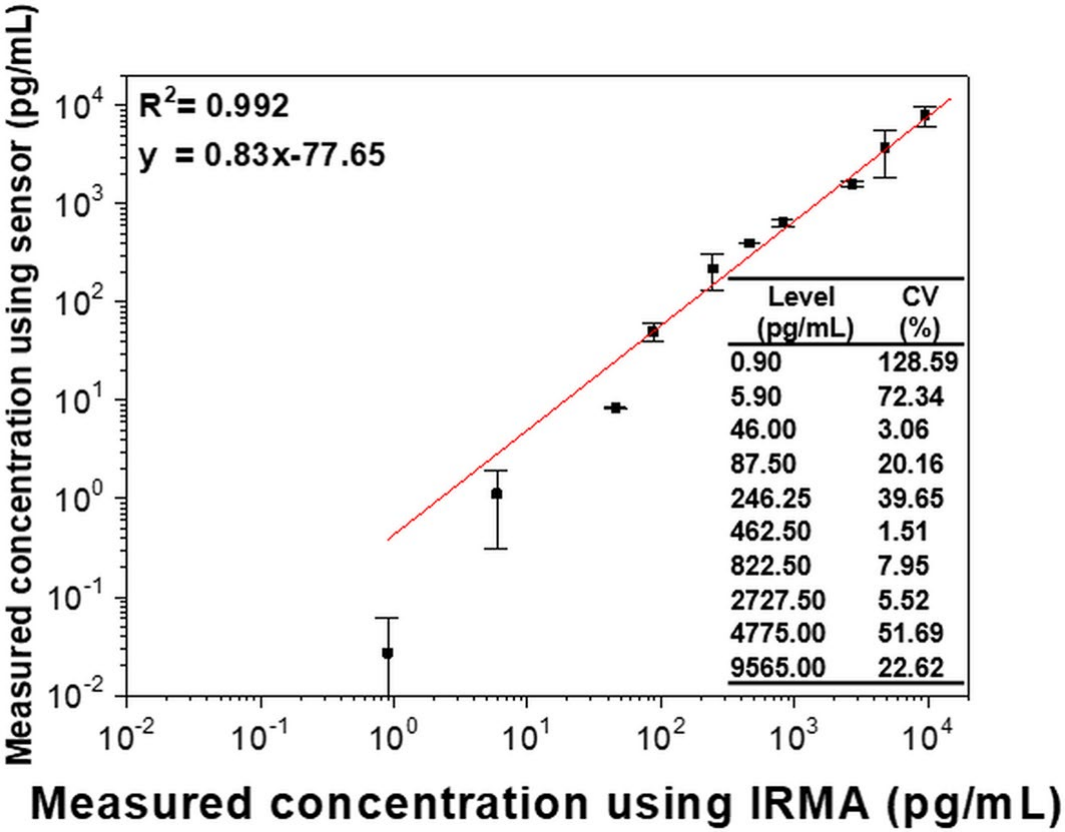
Correlation test between obtained Tg concentrations by the FO LSPR sensor chip and commercial kit.

To validate and expand the sensor chip, Tg in patient serum was detected. Samples taken from a total of 10 patients were measured a total of 30 times, and the results were compared with the values measured in the commercial IRMA instrument (GAMMA-10, Shin Jin Medics). In Fig. 7, the x-axis value is the Tg concentration measured using the IRMA kit, whereas the y-axis is the Tg level detected by the proposed sensor. The R^2^ between the two Tg concentrations by IRMA and our sensor was 0.992, which indicates a high correlation between the two variables. The slope of the linearly fitted graph in Fig. 7 was slightly less than 1 because the Tg levels measured by the proposed sensors were lower than those measured by IRMA at low concentrations. This may be because of an increase in the dilution rate in samples with low concentrations^40^. In the serum sample testing, the LOD was calculated as 21.18 pg/mL, which is several hundred times higher than the LOD measured in the DI buffer described in “Tg detection” section. The degraded LOD is presumed by a phenomenon known as the matrix effect, which can reduce the performance of the sensor. The matrix effect is caused by the nonspecific binding of various matrix components constituting biological fluids^41^. Also, an average CV of 19.23% was observed at concentrations above the LOD, which is satisfactory in biosensing applications^42^. The CV is widely used in analytical chemistry to indicate the reproducibility of an assay. Further enhancement of the performance can be achieved by modifying the sensor surface. Quantification of target molecules in serum is important for the early diagnosis of recurrence and prediction of disease progression and recovery.

## Conclusion

In this study, we present the fabrication and characterization of a fiber optic localized surface plasmon resonance sensor for Tg immunoassay. The concentration of serum Tg after total thyroidectomy is a sensitive indicator for recognizing residual thyroid cancer and recurrence. Nowadays, a relatively long time (1 day or more) may be used for Tg immunoassays. A rapid and highly sensitive FO LSPR sensor system for Tg can reduce pain and burden on patients by early detection of residual cancer and recurrence. The proposed FO LSPR sensor combined with MFC showed a LOD of 93.11 fg/mL and only 10 min were consumed for the measurement of Tg. Compared to conventional techniques, the LOD of FO LSPR is approximately 100 times lower. The consumption time of FO LSPR is approximately 6–120 times shorter than that of IRMA, IeRA, and CLIA. A more sensitive and rapid Tg measurement is established. Additionally, interference with other antigens was not confirmed, and the values of the test samples with unknown concentrations could be estimated with fair accuracy. When the proposed sensor was applied to detect Tg in the patient’s serum, it showed a high correlation with a commercial diagnostic kit. Consequently, the developed sensor chip could be a valuable tool for the sensitive monitoring of DTC.

## Supplementary Information

Supplementary Information.
