# Supplementary material for: Design and validation of fiber optic localized surface plasmon resonance sensor for thyroglobulin immunoassay with high sensitivity and rapid detection
Source: Sci Rep. 2021 Aug 6;11:15985. doi: 10.1038/s41598-021-95375-y (PMC8346482; doi:10.1038/s41598-021-95375-y)
Supplement: Supplementary file 1 — Supplementary Information. [file 41598_2021_95375_MOESM1_ESM.docx]

**Supplementary information**

**Design and validation of fiber optic localized surface plasmon resonance sensor for thyroglobulin immunoassay with high sensitivity and rapid detection**

Hyeong-Min Kim^1^, Dae Hong Jeong^2^, Ho-Young Lee^3,*^, Jae-Hyoung Park^1,*^ & Seung-Ki Lee^1,*^

^1^Department of Electronics and Electrical Engineering, Dankook University, Yongin 16890, South Korea

^2^Department of Chemistry Education, Seoul National University, Seoul 08826, South Korea

^3^Department of Nuclear Medicine, Seoul National University Bundang Hospital, Seongnam 13620, South Korea

*Corresponding author.

E-mail: debobkr@gmail.com; [parkjae@dankook.ac.kr](mailto:parkjae@dankook.ac.kr); [skilee@dankook.ac.kr](mailto:skilee@dankook.ac.kr)


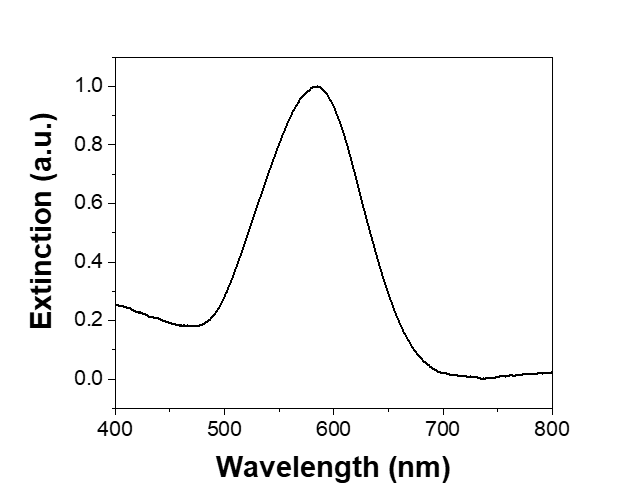


**Figure S1.** UV-Vis spectrum of the synthesized colloidal solution of gold nanoparticles.


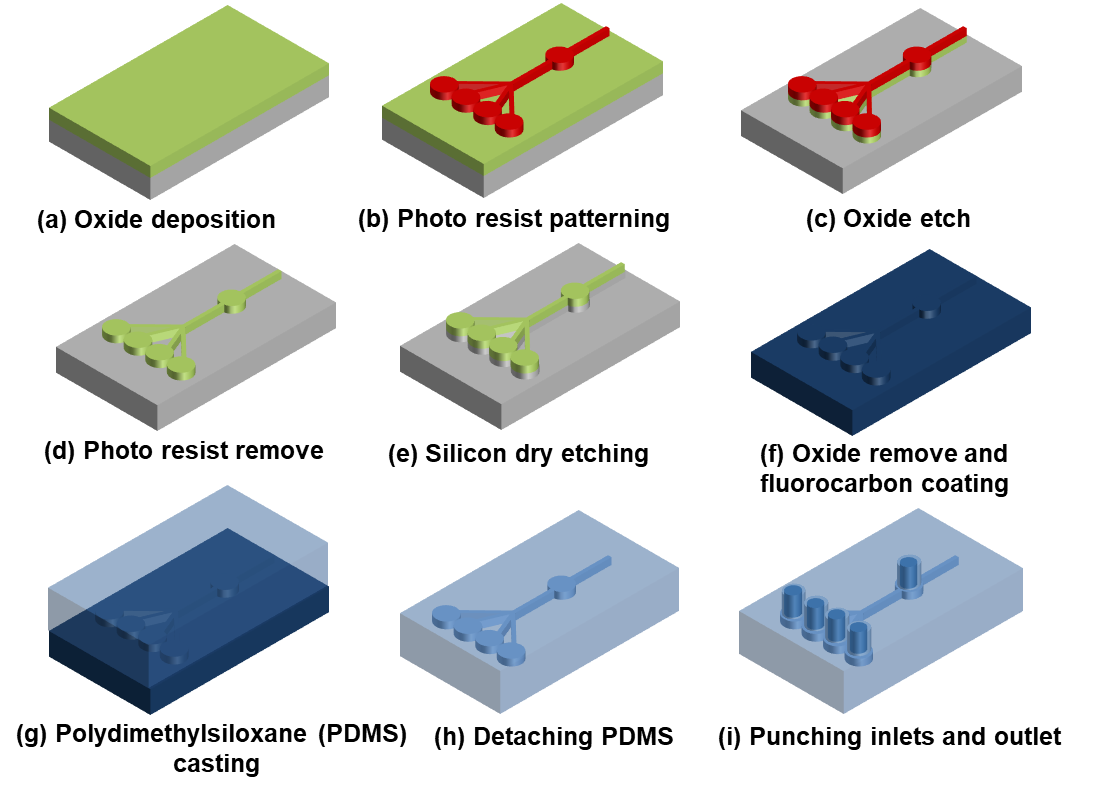


**Figure S2.** Manufacturing process of the microfluidic channel.


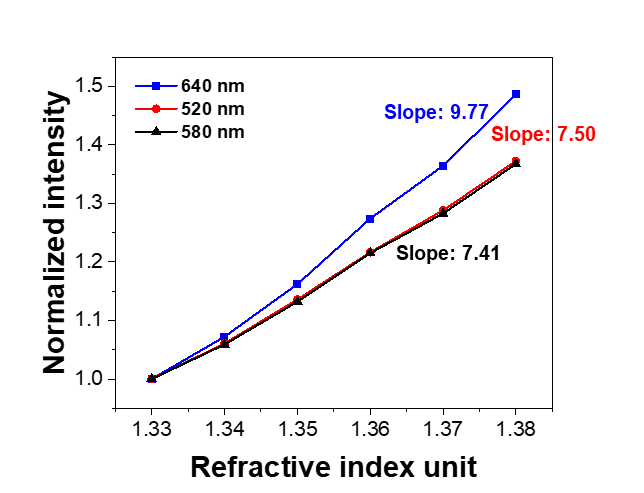


**Figure S3.** Refractive index responses at different wavelengths in the spectrum excited by white light.


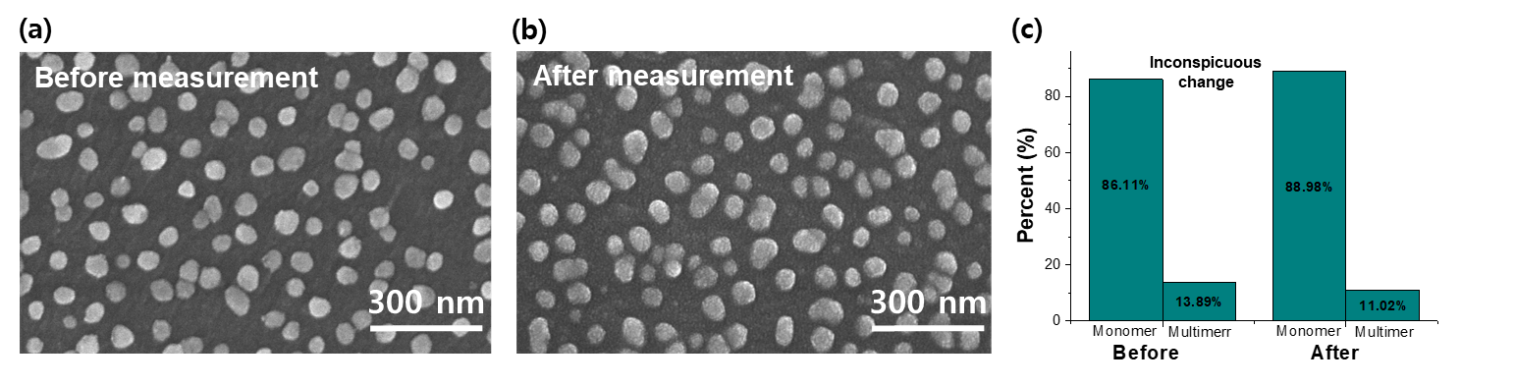


**Figure S4.** Cross-sectional pictures of the optical fiber before and after the experiment and histogram of nanoparticle distribution: images of fiber optic surface (a) before and (b) after measurement and (c) distribution of nanoparticles such as monomer and multimer.
